# Analysis and process evaluation of metal dopant (Zr, Cr)-promoted Ga-modified ZSM-5 for the oxidative dehydrogenation of propane in the presence and absence of CO_2_†

**DOI:** 10.1039/d2ra08235g

**Published:** 2023-04-06

**Authors:** Abbas Jawad, Sura Ahmed

**Affiliations:** a Midland Refineries Company MRC/AL Daura Refinery Company/Training and Development Division/Baghdad Iraq ajd5d@mst.edu; b Midland Refineries Company MRC/AL Daura Refinery Company/Maintenance Board/Baghdad Iraq bearn_bearn2020@yahoo.com

## Abstract

A systematic study of the comparative performances of 4% Ga-, 4% Zr-, and 1% Cr-impregnated H-ZSM-5 catalysts for oxidative dehydrogenation of propane in the presence and absence of CO_2_ is presented. It was found that methane, ethene, propene, butene, pentene, and BTX are the major products from all of these catalysts at various reaction temperatures (400–550 °C), WHSV, 4 kg_cat_ s mol_C_3_H_8__^−1^ and feed gas, C_3_H_8_/N_2_ = 2.5/97.5 and C_3_H_8_/CO_2_/N_2_ = 2.5/5/92.5; flow rate, 75 mL min^−1^ under atmospheric pressure for 10 h. The combination of material characterization and catalytic testing revealed that Ga-, Zr-, and Cr-doped H-ZSM-5 are excellent catalysts for this process, helping to achieve around 61% CO_2_ conversion. The co-doped Ga/H-ZSM-5 catalysts significantly enhanced the activity (65% propane and 61% CO_2_ conversion at 550 °C) among all the tested catalysts, with approximately 100% total selectivity 62% towards BTX and 26% towards propene, but with lower selectivity for methane, ethene, and pentene (*i.e.*, light hydrocarbons). TPR profiles indicated that the redox cycle between Cr(iii)O_6_ and Cr(vi)O_4_ played an important role in the dehydrogenation of C_3_H_6_ over Cr/Zr–Ga/H-ZSM-5. CO_2_ could oxidize a part of the Cr(iii) species to Cr(vi) species under the reaction conditions used.

## Abstract 2

A systematic study of the comparative performances of 4% Ga-, 4% Zr-, and 1% Cr-impregnated H-ZSM-5 catalysts for oxidative dehydrogenation of propane in the presence and absence of CO_2_ is presented.

## Graphical abstract

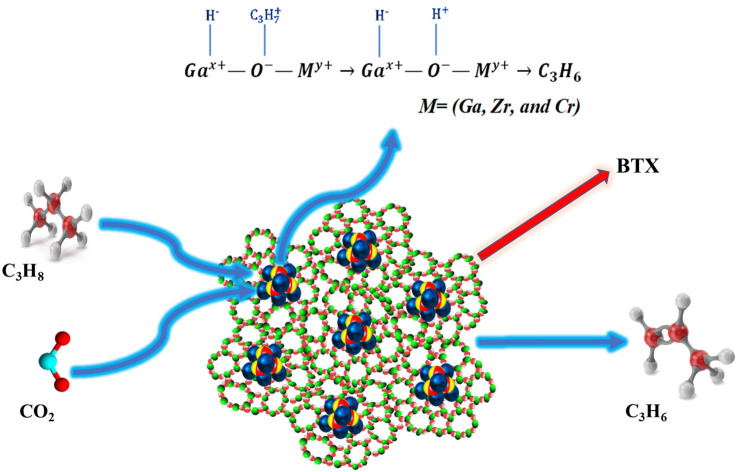

## 1. Introduction

There is a large and growing worldwide demand for short-chain alkenes, particularly ethene and propene. It is estimated that production of these two chemical building blocks exceeded 300 M tonnes in 2020.^1^ Propene is one of the principal raw materials used in the petrochemical industry, mainly in polymer and rubber manufacture; the growth in demand for propane requires new production methods since the two main commercial processes (steam cracking of naphtha or liquid petroleum gas, and fluid catalytic cracking (FCC) of heavier oil fractions) have already essentially been optimized for propene production.^2^ Consequently, there has been intensive investigation of alternative routes that require reduced energy expenditure, such as the transformation of alkanes to the corresponding alkenes, as well as other new technologies. Selective dehydrogenation of propane to propene is one of the major challenges faced in the production of precious feedstocks. An alternative route that has recently received attention is the non-oxidative catalytic dehydrogenation of propane (CDP), which operates at high temperatures (above 527 °C) in order to overcome thermodynamic restrictions, with consequent catalyst deactivation due to propane/propene cracking and coke deposition, leading to decreased propene selectivity and yield.^3^ The oxidative dehydrogenation of propane (ODP) is attractive because the reaction is exothermic, and it can proceed at lower temperatures, suppressing coke formation. However, this process suffers from a significant loss of propylene selectivity due to the overoxidation of propane to carbon dioxide in the reaction. Handling the potentially explosive oxygen-containing mixture is also difficult. This drastically affects the olefin yield due to the low concentrations of propane employed. In order to circumvent these problems and improve process selectivity and yield, carbon dioxide has been proposed as a mild oxidant to replace oxygen.^4^ Technological advances, with removes hydrogen produced from propane dehydrogenation and shifts the dehydrogenation equilibrium towards the propylene formation and increases propene yield, can be achieved using higher propane concentrations in the process and/or coke gasification by CO_2_ through the reverse Boudouard reaction (CO_2_ + C ↔ 2CO; Δ*H*_298 K_ = +172 kJ mol^−1^). Moreover, the side benefit of the new process is that CO_2_ is converted to a useful co-product, carbon monoxide. Moreover, carbon dioxide can promote the dehydrogenation reaction by the reversed water-gas shift reaction over supported ex. Cr_2_O_3_ and Ga_2_O_3_ catalysts.^5^ Zeolites are a class of microporous crystalline materials that have found widespread use in the petrochemical industry. ZSM-5, a typical MFI-type zeolite, is a good catalyst or catalyst support^6^ because of its unique properties, including tridimensional micropore structure, high surface area, high thermal and hydrothermal stability, special molecular sieving, and shape-selective capabilities. Our particular interest is in exploring the applications of ZSM-5-supported oxide catalysts in the oxidative dehydrogenation of propane in the presence of carbon dioxide (ODPC).^7^ Among them, considerable attention has been dedicated to chromium and gallium oxides, which are the most active, selective, and stable in the presence of CO_2_. It is well known that Ga-loaded ZSM-5 is a good catalyst for the aromatization of ethane and propane. The process is regarded as a bifunctional mechanism in which gallium oxide is responsible for catalyzing the dehydrogenation of propane to propene in the presence of CO_2_ while Brønsted acid sites catalyze the oligomerization step. The high propene yield and superb stability were attributed to the decrease in the number of acid sites with medium to strong strength on the catalysts, which results in the suppression of side reactions such as oligomerization, cyclization, cracking, and aromatization.^8^ Chromium is a very commonly used as an oxidative and dehydrogenative metal in various commercial processes.^9^ Fu *et al.*^10^ reported the aromatization of propane over a chromium-modified ZSM-5 prepared by solid state reaction. This catalyst showed high selectivity for propylene. Williams *et al.*^11^ observed that compared to H–Y, the high activity of H-USY can be ascribed to the enhancement of the hydride transfer reaction while also introducing the oligomeric cracking mechanism into the reaction network, accelerating coke formation and catalyst deactivation. It has been noticed that Zr incorporation over HZSM-5 can enhance hydride transfer reactions in *n*-pentane catalytic cracking,^12^ and enhance light olefin yields in naphtha catalytic cracking.^13^ In this paper, a series of Cr-, Zr-, and Ga-doped H-ZSM-5 catalysts were prepared through the wet impregnation method. The effects of Cr, Zr, and Ga on the structural and adsorption properties were systematically determined. The impact of surface components on optimal composition was studied in particular. According to the results, this research reported the investigation of different metal oxides as bi-metallic and tri-metallic supported on H-ZSM-5 catalysts of physicochemical characterization, which greatly alters the adsorption properties of two reactants (C_3_H_8_ and CO_2_) during oxidative dehydrogenation of propane in the presence and absence of CO_2_ and the effect on catalytic performance, propene selectivity and product distributions for propane particularly aromatics (BTX).

## 2. Experimental

### 2.1. Catalyst synthesis

Pristine MFI zeolite powder, in the form of commercial ZSM-5 (CBV 8014, Zeolyst International, nominal Si/Al ratio = 50, NH_4_-form), was chemically altered to the protonic form by calcination at 550 °C for 10 h in static air (ramp rate = 10 °C min^−1^). The transition metal oxide precursors, including Ga(NO_3_)_3_·*x*H_2_O, Cr(NO_3_)_3_·9H_2_O, and Zr(NO_3_)_3_·6H_2_O. oxide were all purchased from Sigma-Aldrich. The introduction of Ga, Zr, and Cr cations was accomplished by wet impregnation in stirred aqueous solutions of the corresponding nitrates (Sigma Aldrich, 99%) and was then dried overnight at 120 °C. Following that, the samples were calcined in a muffle furnace at 550 °C for 6 h (ramp speed = 10 °C min^−1^). The metal loadings for 4% Ga/H-ZSM-5 in weight fraction were set at 4% Ga and 4 wt% Zr for 4% Zr–Ga/H-ZSM-5; and 1% Cr, 4% Zr, 4% Ga, for 1% Cr/4% Zr–Ga/H-ZSM-5 as list in Table 1.

**Table 1:** Identification of abbreviations for H-ZSM-5 dopped with metal oxides (Ga, Zr, and Cr)

Composites	Ga (wt%)	Zr (wt%)	Cr (wt%)
H-ZSM-5	—	—	—
4% Ga/H-ZSM-5	4	—	—
4% Zr–Ga/H-ZSM-5	4	4	—
1% Cr/4% Zr–Ga/H-ZSM-5	4	4	1

### 2.2. Catalyst characterization

Catalytic X-ray diffraction (XRD) patterns were obtained using monochromatic CuKα1 radiation (*λ* = 0.154178 nm) using a PANalytical operating at 30 kV and 15 mA. The XRD pattern was evaluated at a step size of 0.026° from 5° to 50° 2*θ* and rate of 2° min^−1^. X-ray fluorescence (XRF) analysis utilizing the X-Supreme8000 was used to evaluate the chemical composition of the materials in order to calculate the weight percent loading of the metals. The temperature-programmed desorption (NH_3_-TPD) and (CO_2_-TPD) were carried out to investigate the acidic and basic properties of the metal oxide-doped H-ZSM-5 using a Micromeritics 3Flex analyzer. Normally, 100 mg of powder test was placed in a U-shaped fixed-bed reactor, preheated to 530 °C for 1 h, and then cooled to 100 °C. Then, at that point, NH_3_ or CO_2_ gas was introduced to saturate the sample. Gas injection test-carrier He is then used to remove excess NH_3_ or CO_2_. After stabilizing for 1 h, the sample was heated to 800 °C at a rate of 10 °C min^−1^. Online mass spectrometry (MKS) was used to monitor the NH_3_ or CO_2_ desorption profile over a temperature range of 100–800 °C. This was followed by online mass spectroscopy (MKS). H_2_-TPR measurements were carried out in a U-shaped quartz cell using a 5% vol H_2_/He gas with flow rate of 30 cm^3^ min^−1^ at a heating rate of 10 °C min^−1^ up to 800 °C using a Micromeritics 3Flex analyzer. Hydrogen consumption was followed by online mass spectroscopy (MKS) and quantitative analysis was performed by comparison of reduction signal with the hydrogen consumption of a CuO reference. Evaluation of the physical adsorption isotherm of N_2_ was performed on a Micromeritics 3Flex surface analyzer at −196 °C. Textural properties such as surface area, total pore volume, micropore volume, and average pore width were determined using the Brunauer–Emmett–Teller (BET), Barrett–Joyner–Halenda (BJH), and t-plot methods, respectively. The surface topographies were assessed by high-performance field emission SEM on a Zeiss Merlin Gemini microscope. EDS was collected on a Bruker 5030 X-Flash diffractometer using an accelerating voltage of 25 kV. With the use of an EMIA-220V Horiba Carbon–Sulfur Analyzer Model, coke was analyzed on the spent catalyst. The furnace burnt used catalyst at high temperatures for 10–20 mg (tungsten added as a combustion promoter). In order to calculate the carbon content as a percentage of the catalyst's weight, subsequent combustion gas (CO_2_) was run through an infrared analyzer.

### 2.3. Catalyst evaluation

To measure the catalytic activity of the system in relation to propene selectivity, catalytic tests were performed in packed bed stainless steel reactors with an internal diameter of 12.7 mm and a length of 300 mm. The feed gas contained C_3_H_8_/N_2_ = 2.5/97.5% or 2.5% C_3_H_8_/CO_2_/N_2_ = 2.5/5/92.5%, which was injected at a flow rate of 75 mL min^−1^; the flow rate into the reaction regions was controlled *via* a digital mass flow controller (MFC, Brocks Instrument). Nitrogen was used as an internal standard to account for changes in the rate of ethane flow caused by the reaction. In a typical experiment, 300 mg of catalyst (particle size 0.5 nm) was diluted with sand in a ratio of 1 : 4 and placed in the center of the reactor with quartz wool at both ends. The test was evaluated within a temperature range of 400–550 °C at a constant weight hourly space velocity (WHSV), 4 kg_cat_ s mol_C_3_H_8__^−1^. Prior to the reaction, the catalyst was activated at 600 °C under nitrogen (N_2_) flow for 1 h. Next, the sample was reduced at 600 °C for 1 h using 5% H_2_/He. After purging with N_2_ for 5 min, 75 mL min^−1^ of 10% CO_2_/Ar was introduced to reoxidize the catalyst, and a catalytic test was conducted. The reaction products were analyzed online every 30 min *via* a gas chromatogram (SRI 8610C) equipped with a flame ionization detector (GC-FID) connected to a capillary column to detect propene and other hydrocarbons. The thermal conductivity detector (TCD) for H_2_, CO_2_, CO, H_2_O, and hydrocarbons. The effluent line from the reactor to the GC injector was maintained at 110 °C to avoid possible condensation of hydrocarbons.

## 3. Results and discussion

### 3.1. Characterization of the catalyst

#### 3.1.1. XRD and XRF analysis

**Fig. 1:**
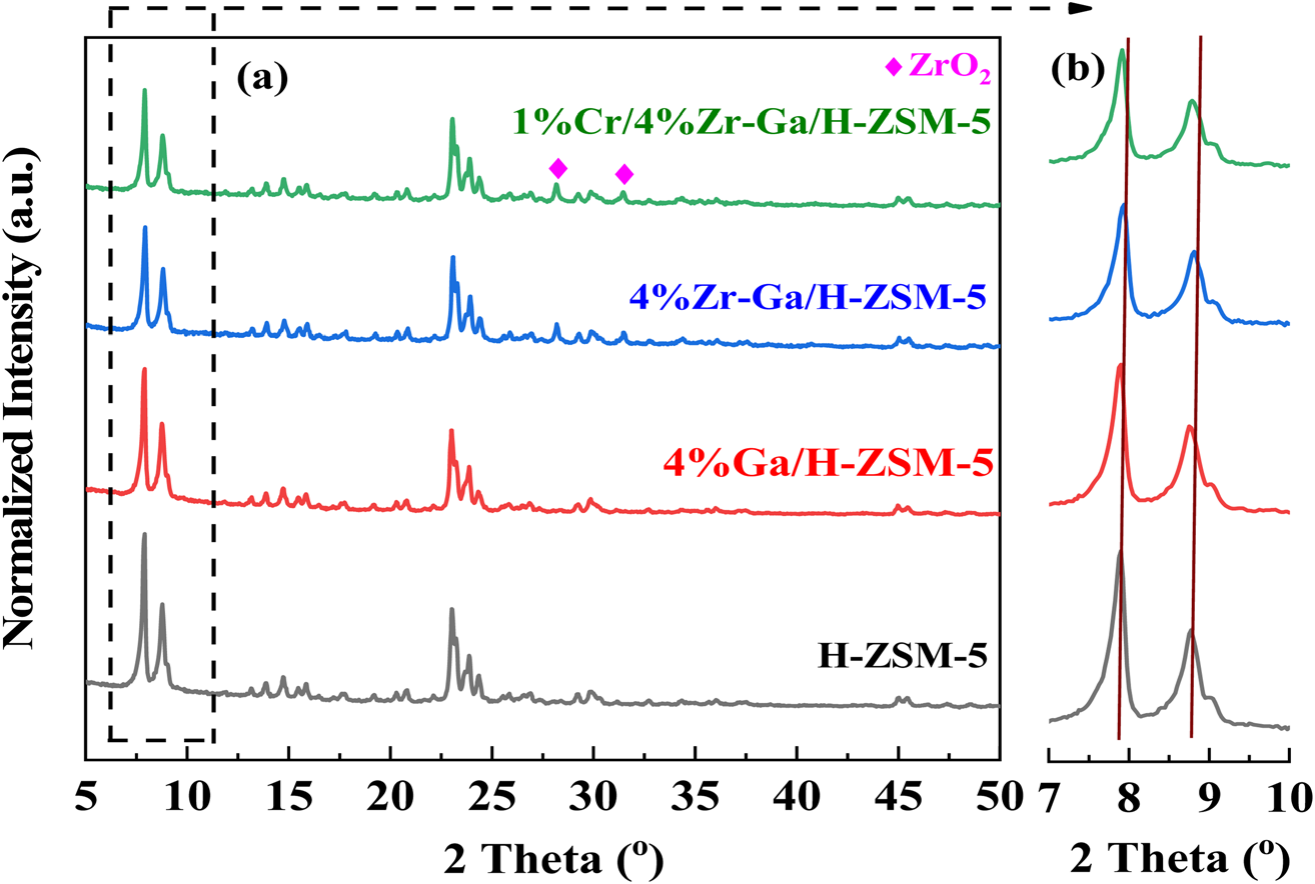
(a) XRD patterns and (b) small angle X-ray diffraction patterns between 7° and 10°.

The XRD patterns of the synthetically doped H-ZSM-5 catalysts are displayed in Fig. 1. The peaks observed at 2*θ* = 7.89°, 8.83°, 23.1°, 23.43°, and 24.0° were attributed to the (101), (200), (501), (341), and (303) planes of the zeolite crystal with an MFI framework. It showed that the H-ZSM-5 framework was well preserved after metal incorporation. Scrutiny of the patterns in Fig. 1a in the range 2*θ* = 28–50° revealed that both samples contained metal oxide. The peaks at 2*θ* = 28.25° and 31.52° in Zr–Ga/H-ZSM-5 can be attributed to ZrO_2_.^14^ No obvious diffraction peaks were found within the angles investigated that corresponded to the gallium and/or chromium species observed in the XRD patterns, indicating that gallium and/or chromium species may be highly dispersed upon the surface of the ZSM-5 zeolite^15^ or aggregated into mini-crystals that are too small (less than 4 nm) to show obvious diffraction peaks.^16^ This is consistent by XRF profiles (refer Table S-1, ESI†). Furthermore, the low metal loading in samples, location of the cations in the ion exchange positions, or dispersion peaks of the parent H-ZSM-5 zeolite might result in possible peak overlap/coincidence. It is generally accepted that the small angle X-ray diffraction (SAXRD) patterns, as shown in Fig. 1b for microporous materials, are highly sensitive to the presence of any particles inside their micropore channel structures, where the intensity and the *d*-spacing of the diffraction peak change accordingly.^17^ Moreover, the height of the diffraction lines of the mesoporous samples is slightly lowered and broadened, which is due to the presence of nanometer-sized crystals, as based on the Scherrer equation.^18^ In order to compare the XRD patterns of these catalysts, the data were normalized with respect to the diffraction peak at 2*θ* = 23.1°. The intensity of the low angle diffraction peaks between 2*θ* = 7–10° was significantly lower with Zr catalyst (Zr–Ga/H-ZSM-5) compared to Ga/H-ZSM-5 and H-ZSM-5 catalysts, which could indicate a strong interaction between the Zr and the Al framework on the catalyst surface, leading to reduced crystallinity.^19^ Due to the impregnation process used in the manufacture of these catalysts, most of the particles probably remain on the outer surfaces of the zeolite and do not spread into the pores of the HZSM-5. Conversely, Ga_2_O_3_ can thermally diffuse inside H-ZSM-5 micropores and anchor to the Brönsted acid sites, as described by Xu *et al.*^20^ and Borry *et al.*^21^ No effect was observed on the dispersion mechanisms between the Zr–Ga/H-ZSM-5 and Cr/Zr–Ga/H-ZSM-5 catalysts, suggesting that Cr addition did not have a major effect on the crystallinity of this catalyst compared to Zr and Ga. This may have been due to differences in Zr and Cr concentrations.^22^ Table S-1† discusses the XRF analysis results in detail. As can be seen from the table, the theoretical and experimental calculations yield nearly identical results; there is a small difference between these numbers, indicating that a number of substances and materials used in this work are accurate, and both the Si/Al ratios and the amount of metal oxides-loading are consistent with what is used during catalyst preparations.

#### 3.1.2. Acidity (NH_3_-TPD) and basicity (CO_2_-TPD)

**Fig. 2:**
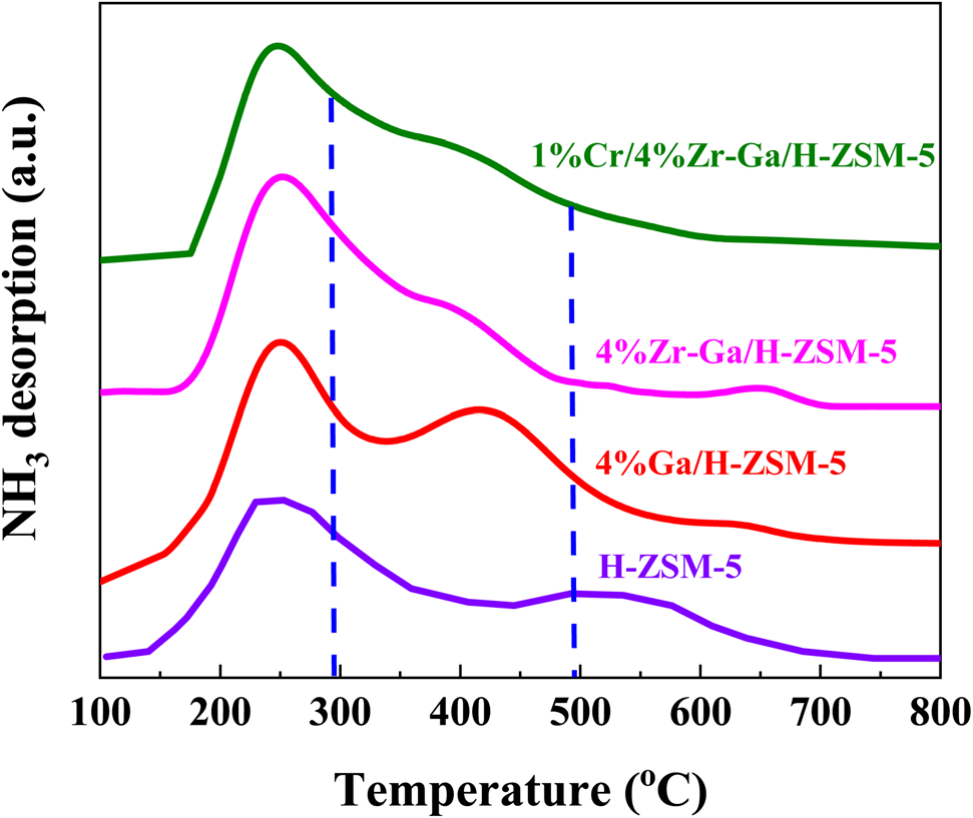
NH_3_-TPD profiles of the bare and Ga-, Zr–Ga-, and Cr/Zr–Ga-doped H-ZSM-5.

The total number of acid sites on the catalysts was measured using ammonia-temperature-programmed desorption (NH_3_-TPD). The NH_3_-TPD profiles of H-ZSM-5 before and after loading with different metal oxides are depicted in Fig. 2 and Table 2. The introduction of (Me = Ga, Zr, and Cr) species on H-ZSM-5 had an effect on the distribution of acid sites and acidity. Within the temperature ranges of 100–297 °C, 297–490 °C, and 490–800 °C, the NH_3_-TPD profiles for all catalysts display three desorption peaks attributed to the weak-medium, medium-strong, and strong acid sites, respectively. The acidity of the bare and metal oxides (Me = Ga, Zr, and Cr) modified H-ZSM-5 catalysts is shown in Table 2. The modified Me/ZSM-5 catalysts have comparable distributions of weak-medium, medium-strong, and reduced strong acid, which works for severe cracking and hydrogen transfer reactions from ring compounds to lower olefins. The maximum of the peaks slightly shifted to the lower temperatures. As ammonia adsorbed at the weak acid sites identified the low-medium temperature peak, which was supposed to effectively alter proton mobility.^23^ The high temperature TPD peak is attributable to firmly bound NH_3_ resulting from NH_3_ bound to zeolite protons. Furthermore, it is proposed that the metal oxide species interacted with the acid sites of ZSM-5, weakening their acid strength.^24^ These results are consisted to STEM and N_2_ sorption analysis, the introduced metal oxides had a well dispersion. It is been reported that, impregnating metals oxides into the bare H-ZSM-5 suppresses the weak and strong acidic sites.^25^ These metal(s) impregnated zeolites are supposed to occupy and fill the pores with non-framework Al, resulting in inaccessible NH_3_ chemisorption and blockage of the strong acidic sites. In addition, it has been shown that suppressing strong acidic sites for H-ZSM-5 inhibits the hydrogen transfer reaction as a result of reduced alkane formation and increased aromatic yield. These changes indicated that the introduced metal species replaced some of the conventional strong acid sites, allowing the zeolites' overall acidity to change to moderate-intensity acidity, which enhanced the adsorption and desorption of NH_3_ molecules and thus play an important role in the selective synthesis of alkane hydrocarbons without polymerization and cracking of products due to mild acid strength,^20,26^ as referred to in the XRD and BET results. Consequently, metal species, (Me = Ga, Zr, and Cr), can react with acidic OH groups to form Me–OH^+^ after being introduced into the zeolite structure. This Me–OH^+^ species accepts more and interacts strongly with the negative charge created by the interior silanol groups. The M–O–Si bonds are created as the new moderate Lewis (M-Lewis) acid sites *via* the following process.ZO–MeOH^+^ + Si–OH → ZO–Me–O–Si

**Table 2:** Summary of calculations for acid site analysis based upon NH_3_-TPD

Catalyst	Desorbed NH_3_ value^a^ (μmol g_cat_^−1^)			Total acid sites
	100–297 °C	297–490 °C	Above 490 °C	
H-ZSM-5	180	149	270	599
4% Ga/H-ZSM-5	190	158	68	416
4% Zr–Ga/H-ZSM-5	248	185	92	525
1% Cr/4% Zr–Ga/HZSM-5	225	230	55	510

a Determined from NH_3_-TPD results.

The novel M-Lewis acid sites accelerate the dehydrogenation of alkenes to aromatics. These sites are more selective to aromatic compounds, particularly BTX, and by regulating the hydrogen transfer process, they limit the synthesis of heavy aromatics, resulting in superior anti-coking ability than Brønsted acidic centers, which is believed to be significantly beneficial to the ODPC or ODP reaction.^27^ The CO_2_-TPD profiles of the various basic catalysts, namely H-ZSM-5, Ga/H-ZSM-5, Zr–Ga/H-ZSM-5, and Cr/Zr–Ga/H-ZSM-5. The CO_2_ desorption rate as a function of desorption temperature is depicted in Fig. S-1.† The TPD profiles of those samples show numerous CO_2_ desorption peaks across a large desorption range, indicating the presence of a variety of basic sites with varying intensities. These desorption peaks may be classified into three zones based on their desorption peaks, namely weak (*T* < 281 °C), medium (281 °C < *T* < 501 °C), and strong basicity (*T* > 501 °C). It can be attributed to the weak basicity derived from surface OH groups,^28^ the medium basicity derived from bidentate carbonates desorbed from Me^2+^–O^2−^pairs,^29,30^ and the strong basicity represents the desorption of strongly-adsorbed hydrogen either on the surface of bulk H-ZSM-5 or other metal oxides surfaces, respectively.^31^ H-ZSM-5 and Ga/H-ZSM-5 catalysts, in particular, exhibited high-intensity peaks in the temperature range (*T* < 281 °C), indicating that the H-ZSM-5 and Ga/H-ZSM-5 catalysts enhanced the number of weak adsorption active sites but did not improve overall adsorption strength. The weak adsorption of CO_2_ over H-ZSM-5 and Ga/H-ZSM-5 catalysts limits its contact with the catalyst sites and thus weakens the performance of reaction. The CO_2_ desorption peaks for Zr-, and Cr-doped Ga/H-ZSM-5 catalysts were shifted to higher temperature, indicating strong interaction of the catalytic active sites with CO_2_. The higher amount of moderate and strong active were formed as shown in Fig. S-1.† The desorption peak at high temperature in the CO_2_-TPD profile can be attributed to the desorption of CO_2_ molecules adsorbed on the oxygen vacancies of metal oxide sites. As a result, the activity of moderate and strong basic sites in Zr- and Cr-doped Ga/H-ZSM-5 catalysts is greater than that of weak basic sites in other oxides. As a result, it is predicted that the moderate and strong basic sites are responsible for the reaction, which increases the production of aromatic hydrocarbons.^32^

#### 3.1.3. Surface redox (H_2_-TPD)

**Fig. 3:**
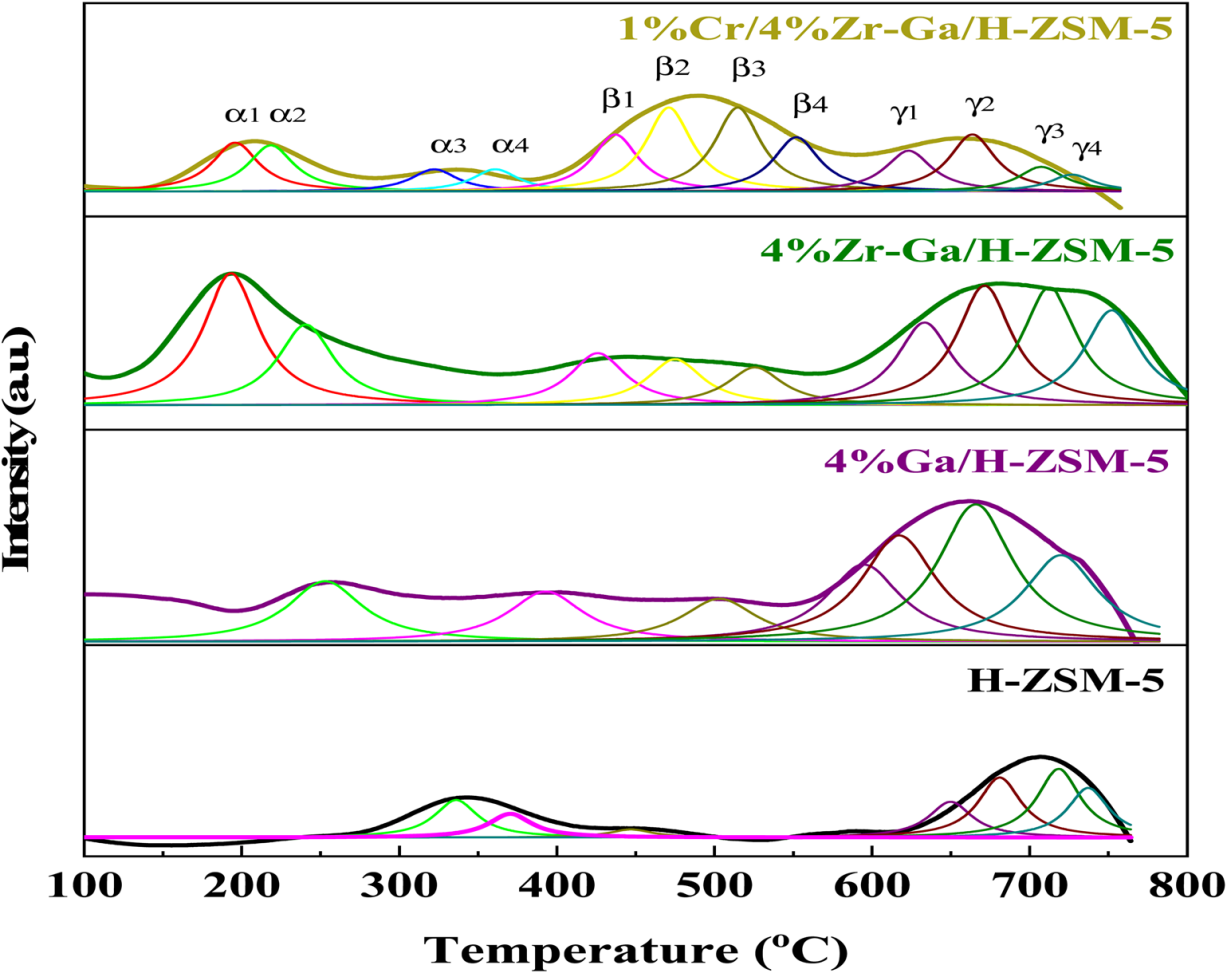
H_2_-TPR profiles with peak fitting of the bare and Ga-, Zr–Ga-, and Cr/Zr–Ga-doped H-ZSM-5.

In order to study the reducibility of the various metal-doped-H-ZSM-5, H_2_-TPR analysis was performed, and the corresponding reduction curves and amount of H_2_ consumption are reported in Fig. 3 while the corresponding relative active site densities were calculated using deconvoluting the peaks by the Gaussian curve fitting method of the H_2_-TPR profiles as gathered in Table 3. The H_2_-TPR profile of the Ga/H-ZSM-5 catalyst consisted of two major low-temperature reduction peaks labeled the α2 and β1 peaks, and five major high-temperature reduction peaks labeled the β3, γ1, γ2, γ3, and γ4 peaks. The low-temperature reduction is associated with the highest number of exchangeable sites, to the reduction of the reminiscent unoxidized Ga^3+^ cations compensating for the negative charge of the zeolite. Similar results were also observed by Brabec *et al.*^33^ Upon Ga introduction to the H-ZSM-5 zeolite, the reduction becomes difficult, and as such the three reduction peaks of the Ga-modified H-ZSM-5 catalysts translate to higher temperatures (centered ∼β3, and γ1, and γ4) as observed in Fig. 3. The principal reason for the increase in the reduction temperature is related to the Ga_2_O_3_ species being highly dispersed onto the H-ZSM-5 zeolite host. Besides, an additional, and new reduction peak centered at ∼γ2 and γ3 for the Ga-modified ZSM-5 catalysts is present. The previous literature^34^ has reported the strong interaction between highly dispersed Ga species and H-ZSM-5 zeolite produced (GaO)^+^(iii) species, while the (GaO)^+^(iii) species could be further reduced to Ga^+^(i). The peak associated with the reduction from (GaO)^+^(iii) to Ga^+^(i) corresponds to the peak at γ2 and γ3, which is in accordance with the XRD results for Ga/H-ZSM-5 in that no Ga_2_O_3_ peaks were found. The Zr–Ga/H-ZSM-5 reduction profile shows reduction temperature peaks labeled as α1, β1, β2, β3, β4, γ1, γ2, γ3, and γ4, where all these peaks correspond to the reduction of the surface zirconium oxide reduction of Zr^4+^ to Zr^3+^,^35^ as shown in Fig. 3. One reduction peak is also observed at α2 that could be due to the reduction of gallium. Furthermore, the presence of Zr^3+^ cations at anion vacancies makes homolytic dissociation of H possible and thus initiates the formation of hydrides according to 2Zr^3+^ + H_2_ ↔ 2Zr^3+^ − H^−^. The reaction gives rise to two types of hydrides, simple (Zr–H) and bridged (Zr–H–Zr). According to Syzgantseva *et al.*, hydrogen dissociates on Zr^3+^ with a neighboring oxygen vacancy (*v*_o_), leading to formation of Zr–H hydrides and the transformation of Zr cations into Zr^4+^ species.^36,37^ The reduction profile peaks of the Cr/Zr–Ga/H-ZSM-5 catalyst consist of the reduction temperature peaks labeled α1, α2, β1, β2, β3, β4, γ1, γ2, γ3, and γ4, as observed in Fig. 3. The α1 and α2 reduction peaks may be due to the reduction of both zirconium and gallium species and subsequently to the reduction in Cr^5+^ to Cr^3+^, and the Cr^3+^ to either Cr^2+^ or to Cr^0^ reduction peaks.^38^ It is suggested that Cr reduction is dominant over that of the zirconia and gallium species in the Cr/Zr–Ga/H-ZSM-5 catalyst, which may be due to the coverage of zirconia and gallium by the chromium ions. However, H_2_ consumption peaks for zirconia and gallium species are shifted to lower temperatures in the Zr–Ga/H-ZSM-5 catalyst on addition of Cr_2_O_3_. This finding agrees with the results of Thirupathi *et al.*^39^

**Table 3:** Summary of calculations for H_2_ consumption from the H_2_-TPR profiles

Catalyst	H_2_ consumption value^a^ (μmol g^−1^)												
	Peak fitting results												Total H_2_-consumption (α1—γ4)
	α1	α2	α3	α4	β1	β2	β3	β4	γ1	γ2	γ3	γ4	
H-ZSM-5	—	—	281	2	—	—	2	—	3	71	72	64	495
4% Ga/H-ZSM-5	—	3	—	—	12	—	43	—	371	433	521	436	1820
4% Zr–Ga/H-ZSM-5	238	239	—	—	23	30	37	—	275	355	342	346	1885
1% Cr/4% Zr–Ga/HZSM-5	37	40	21	20	90	118	1 29	114	66	65	37	21	759

a Determined from H2-TPR results.

#### 3.1.4. Textural properties (N_2_ physisorption)

**Fig. 4:**
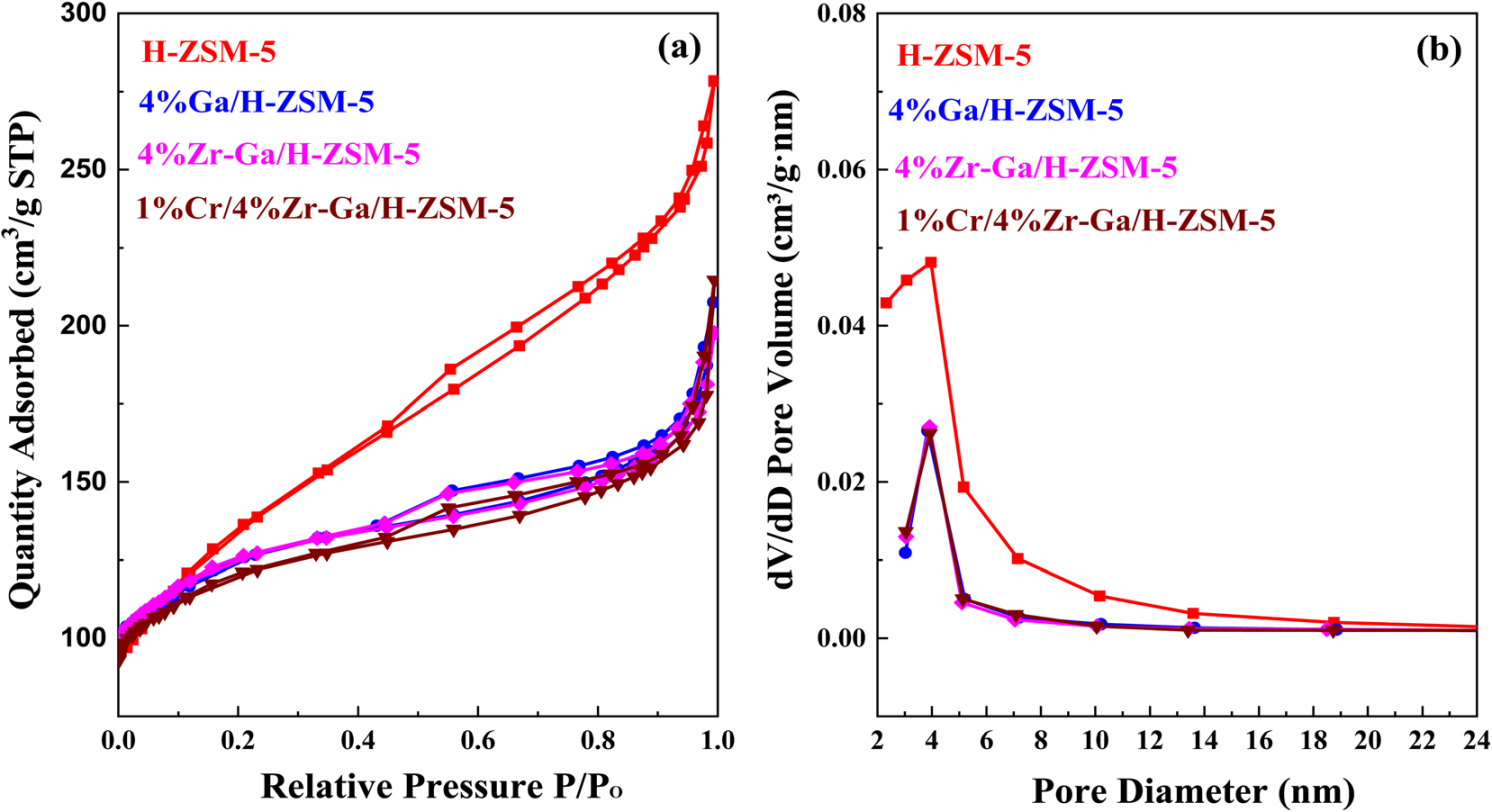
(a) N_2_ physisorption isotherm and (b) the BJH pore size distributions.

N_2_ physisorption isotherms of the as-prepared samples are shown in Fig. 4a, with the corresponding pore size distribution shown in Fig. 4b. All isotherms exhibited a combination of a type IV isotherm with an H3-type hysteresis loop,^40^ with a hysteresis loop at relative higher pressure than *P*/*P*_0_ > 0.4, implying the existence of both micropores and mesopores. These hysteresis loops are usually associated with the capillary filling and condensation of N_2_ within the homogeneous slit-shaped intercrystalline mesopores formed by the aggregation of nanosized zeolite crystals. The textural properties of all metal-doped H-ZSM-5 zeolites are summarized in Table 3. The BET surface area decreased from 470 m^2^ g^−1^ to 417, 415, and 398 m^2^ g^−1^ for H-ZSM-5, Ga/H-ZSM-5, Zr–Ga/H-ZSM-5, and Cr/Zr–Ga/H-ZSM-5, respectively. The lower surface area observed for the impregnated specimens was attributed to partial pore blockage (mesopores in their structure) as a result of the preparation procedure. Similarly, the pore volume is also reduced from 0.43 cm^3^ g^−1^ to 0.32, 0.31, and 0.33 cm^3^ g^−1^ for H-ZSM-5, Ga/H-ZSM-5, Zr–Ga/H-ZSM-5, and Cr/Zr–Ga/H-ZSM-5, respectively. It should be noted here that the slight change in microporous volume, calculated from the t-plot method, indicated that the decline in the total pore volume mainly gives rise to reduction in the mesopore volume. Otherwise, the Ga_2_O_3_, ZrO_2_, and Cr_2_O_3_ particulate could have undergone additional binding to the zeolite clusters. Furthermore, their pore size distributions which are beyond to calcined H-ZSM-5, and the position of peaks are in the range of 2.3–5.8 nm. Thus, the mesoporous structure of the calcined H-ZSM-5 is successfully preserved after impregnation with metal oxide and high-temperature calcination. A narrow and uniform pore size distribution can be obtained for the catalysts from the sharpness of the initial step (at *P*/*P*_0_ = 0.05–0.30), whereas a broad pore size distribution can be observed in the mesoporous region with a primary pore width of 4 nm,^41^ as shown in Fig. 4b. The crystallite sizes of the specimens were also corrected by applying the Debye–Scherrer equation from which the H-ZSM-5 particle size was determined to be less than 25 nm, as indicated in Table 4. The results showed that the average size of the crystal was on the nanometer scale and the addition of metal oxide nanoparticles slightly reduced the size of the crystallite. These results are in good agreement with the previous literature.^42,43^ The active metal surface area (ASA) of a metal increase in proportion to the metal oxides (Zr, Cr) added. Based on the results of the average crystal size, the Ga-dispersion calculation is 22–42%, as shown in Table 3, which indicate the formation of highly metal Ga particles. The high dispersion of the Ga nanoparticles is a result of the homogenous distribution of Ga species in the (Mga/H-ZSM-5) O, [ M = Zr and Cr] calcined product. This is confirmed by XRD profiles (refer Fig. 1) and XRF results (refer Table S-1†).

**Table 4:** Characteristic properties of metal-doped H-ZSM-5 zeolite

Catalyst	*S*_BET_ a (m^2^ g^−1^)	*S*_micro_ b (m^2^ g^−1^)	*S*_ext_ b (m^2^ g^−1^)	*V*_total_ c (cm^3^ g^−1^)	*V*_micro_ b (cm^3^ g^−1^)	*V*_meso_ d (cm^3^ g^−1^)	Pore size^e^ (nm)	*D*_p_ f (nm)	ASA^g^ (m^2^ g^−1^)	*D*%^h^
H-ZSM-5	470	248	222	0.43	0.13	0.30	4.44	21	—	—
4% Ga/H-ZSM-5	417	201	216	0.32	0.10	0.22	5.07	20	9	22
4% Zr–Ga/H-ZSM-5	415	224	191	0.31	0.10	0.21	5.85	19	10	25
1% Cr/4% Zr–Ga/H-ZSM-5	398	211	186	0.33	0.10	0.23	5.80	19	18	42

a Estimated by analyzing nitrogen adsorption data at −196 °C in a relative vapor pressure ranging from 0.05 to 0.30.

b Estimated by the t-plot method.

c Total pore volume was estimated based on the volume adsorbed at *P*/*P*_0_ = 0.99.

d Estimated by subtracting *V*_meso_ = *V*_total_ − *V*_micro_.

e Estimated by BJH desorption average pore diameter.

f Estimated by Debye–Scherrer equation of XRD.

g Estimated by [total number of surface metal atoms] × [cross-section area of active metal].

h Estimated by moles of metal on surface sample/moles of total metal present in sample.

#### 3.1.5. SEM and EDX elemental mapping

**Fig. 5:**
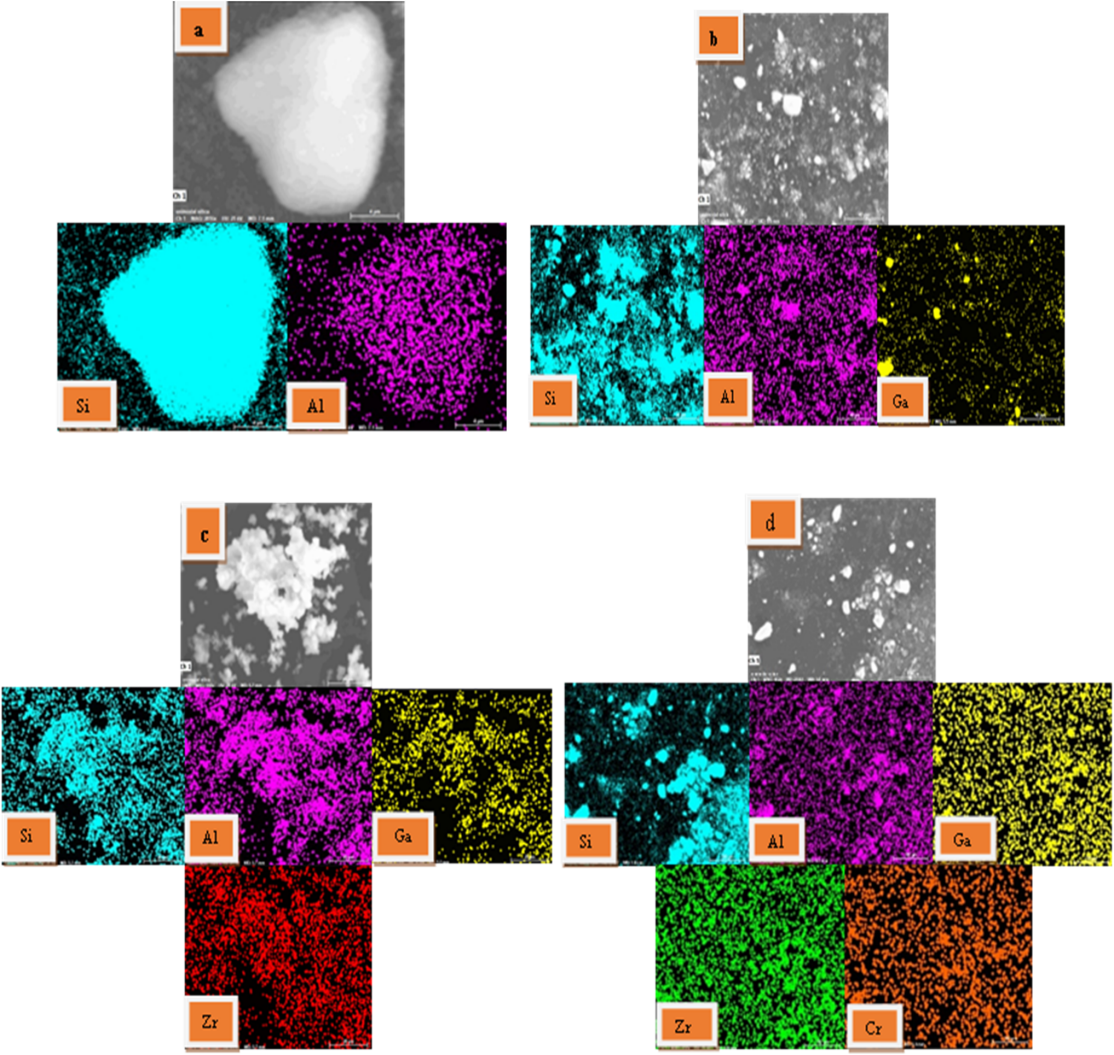
The SEM and EDX diagrams and the elemental mapping images of the synthesized (a) H-ZSM-5; (b) 4% Ga/H-ZSM-5; (c) 4% Zr–Ga/H-ZSM-5; and (d) 1% Cr/4% Zr–Ga/H-ZSM-5 catalysts.

In order to investigate the dispersity of Ga, Zr, and Cr species over the H-ZSM-5 zeolite based-catalyst surface, SEM imaging and EDX elemental mapping were conducted on the H-ZSM-5, 4% Ga/H-ZSM-5, 4% Zr–Ga/H-ZSM-5, and 1% Cr/4% Zr–Ga/H-ZSM-5 samples and are listed in Fig. 5. It is found that all of the elements used for synthesis of catalysts are present in their EDX spectra. These profiles obviously manifest the homogenous distribution of the Ga, Zr, and Cr elements within their corresponding samples. It is noteworthy homogeneous dispersity as well as a fine size of metal oxides particles are key factors governing the catalytic activity, it leads to the appropriate availability of the catalytic active sites to the reactants which results in a high catalytic efficiency.^27^ This finding an agreement with XRD and XRF results (refer Fig. 1 and Table S-1†), respectively.

### 3.2. Catalytic performance

#### 3.2.1. Propane dehydrogenation towards propene

**Fig. 6:**
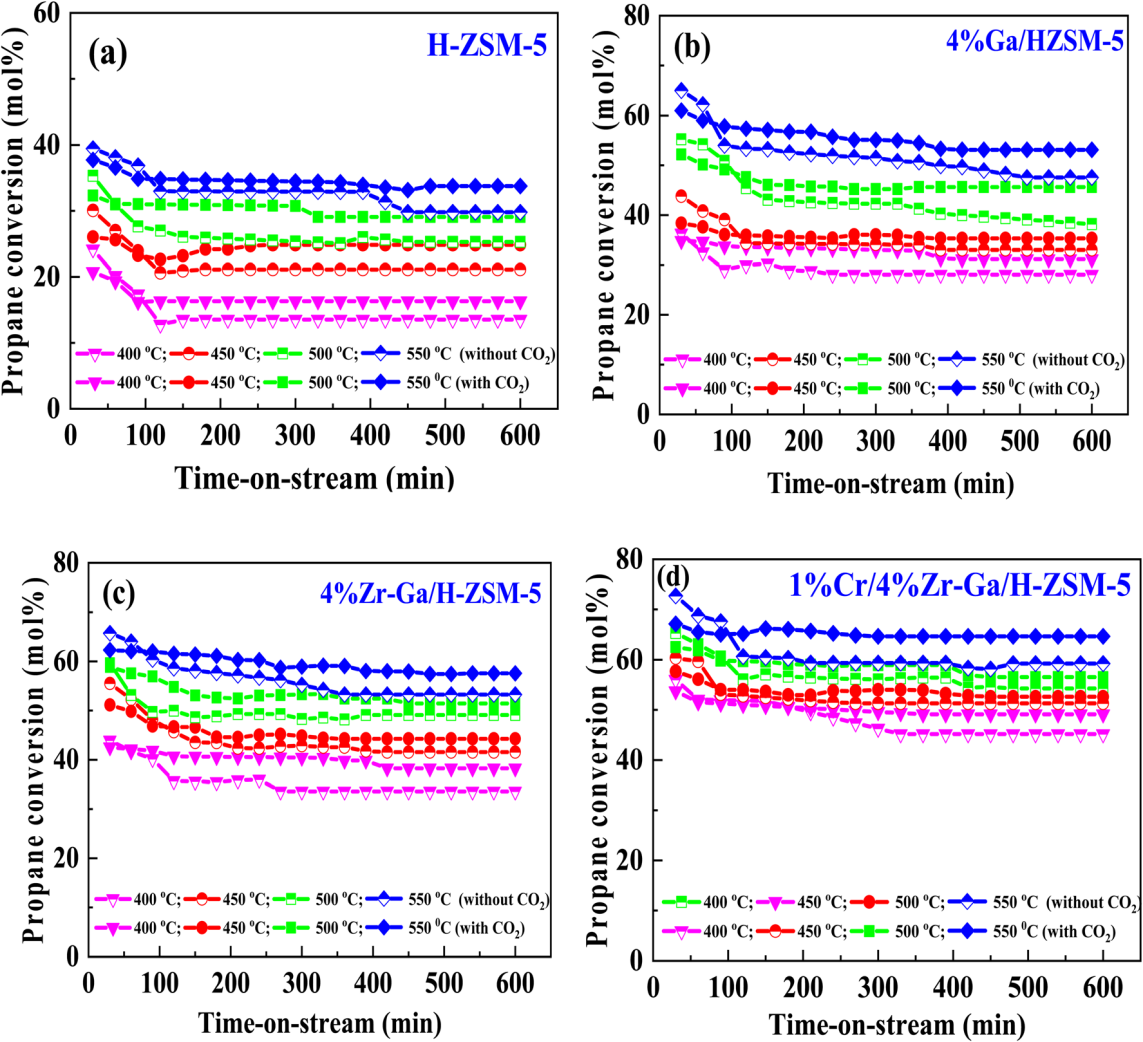
Catalytic performance of (a) H-ZSM-5; (b) 4% Ga/H-ZSM-5; (c) 4% Zr–Ga/H-ZSM-5; and (d) 1% Cr/4% Zr–Ga/H-ZSM-5 catalysts. Reaction conditions: pressure, 1 bar; feed gas, C_3_H_8_/N_2_ = 2.5/97.5 and C_3_H_8_/CO_2_/N_2_ = 2.5/5/92.5; flow rate, 75 mL min^−1^, WHSV, 4 Kg cat s mol_C_3_H_8__^−1^.

**Fig. 7:**
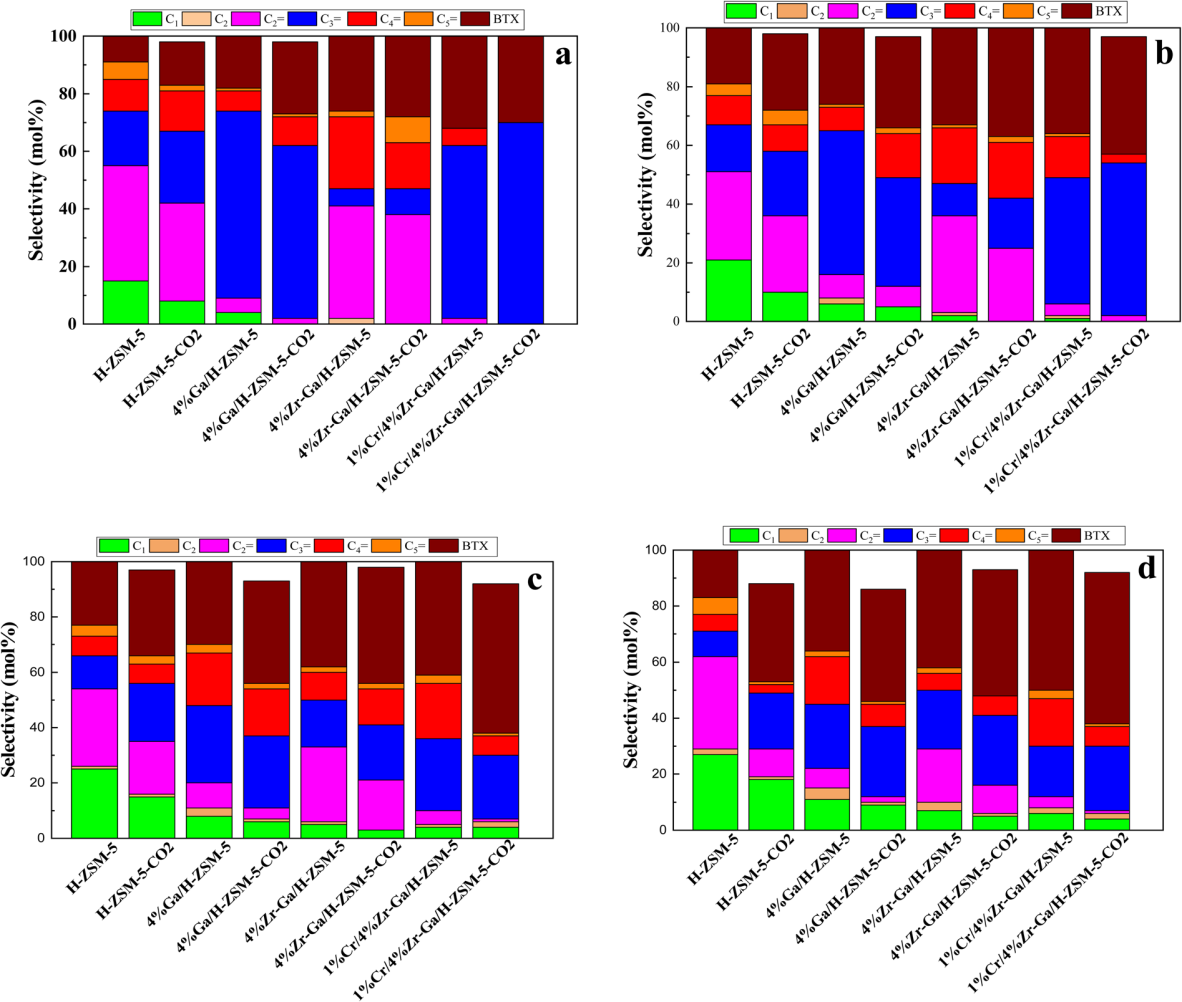
Product distribution of bare and metal-doped H-ZSM-5 zeolites at (a) 400 °C, (b) 450 °C, (c) 500 °C, and (d) 550 °C. Reaction conditions: pressure, 1 bar; feed gas, C_3_H_8_/N_2_ = 2.5/97.5 and C_3_H_8_/CO_2_/N_2_ = 2.5/5/92.5; flow rate, 75 mL min^−1^; WHSV, 4 kg_cat_ s mol_C_3_H_8__^−1^.

**Fig. 8:**
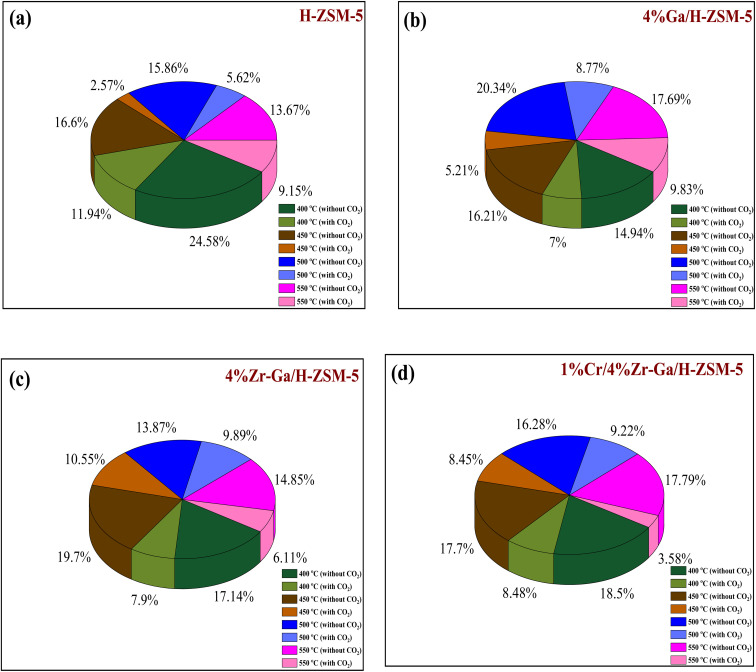
Deactivation factor of the bare and metal-doped H-ZSM-5 zeolites at (a) 400 °C, (b) 450 °C, (c) 500 °C, and (d) 550 °C. Reaction conditions: pressure, 1 bar; feed gas, C_3_H_8_/N_2_ = 2.5/97.5 and C_3_H_8_/CO_2_/N_2_ = 2.5/5/92.5; flow rate, 75 mL min^−1^; WHSV, 4 kg_cat_ s mol_C_3_H_8__^−1^.

The results from initial catalytic evaluation of the H-ZSM-5 supported with metal oxides at the four different temperatures (400, 450, 500, and 550 °C) at atmospheric pressure in both the presence and absence of CO_2_ with a WHSV of 4 kg_cat_ s mol_C_3_H_8__^−1^ for 10 h. The carbon balance contains he products obtained from the propane conversion over the investigated catalysts were mainly paraffins (methane (C_1_) and ethane (C_2_)), light olefins (ethene (C_2_=)), propene (C_3_=), butene (C_4_=) and pentene (C_5_=), aromatics, BTX (benzene, toluene, and xylene) are displayed in Fig. 6, 7 and Table 5. The conversion of propane in presence/absence of CO_2_ increases with increasing reaction temperature (400, 450, 500, and 550 °C) while the selectivity for propene gradually drops, as shown in Fig. 6, 7, Tables 6, and S-2.† At the same time, by-product selectivity (*i.e.*, methane and ethane, *etc.*) increases with increasing temperature, indicating the facilitation of side reactions such as cracking and hydrocracking at high temperatures. In addition, increasing reaction temperature enhances the production of useful by-product, H_2_ and CO, as shown in Table 6 and Table S-2, ESI.† The H-ZSM-5 catalyst performance (Fig. 6a and 7) was further significantly increased with regard to propane conversion and propene selectivity in the presence of CO_2_ compared to its absence CO_2_ (34% *vs.* 32% and 20 *vs.* 9) at 550 °C. The product distribution over H-ZSM-5 during the ODP reaction in the presence and absence of CO_2_ is gathered in Fig. 7. On the other hand, a Ga/H-ZSM-5 catalyst showed a higher conversion of propane and propene selectivity (54% *vs.* 48% and 25% *vs.* 23%) in the presence of CO_2_ and its absence compared to H-ZSM-5, as shown in Fig. 6b and 7. This indicates that gallium incorporated into H-ZSM-5 reduces the concentration of Brønsted acid sites, increases the number of Lewis acid sites, and that there are significant concentrations of strong Lewis acid sites. These results are in agreement with those of Choudhary *et al.*,^44^ where propene selectivity was estimated to be about 15% at this conversion level. The improved performance in these Ga-MFI samples with MPS is likely due to the lower concentration of Brønsted acid sites, as exemplified in gallosilicate was synthesized, namely MG11 for which 3-mercaptopropyl-trimethoxysilane/Ga ratio in the synthesis gel was 1.1, since it is believed that Brønsted acid sites are active in the oligomerization and cyclization steps of alkane aromatization and, subsequently, the formation of coke.^45^ During the oxidative dehydrogenation of propane in the presence of the CO_2_ over Ga/H-ZSM-5, Ga species catalyze the dehydrogenation of propane and intermediates, thus promoting the conversion of propane. Brønsted acid sites are responsible for the oligomerization, cyclization, and cracking of olefins, while Lewis acid sites catalyze the dehydrogenation reaction.^46^ Therefore, the low selectivity of Ga/H-ZSM-5 cracking products is due to the apparent reduction in number of the Brønsted acid sites observed from the NH_3_-TPD profile (see Fig. 2 and Table 2). Therefore, the gallium species and the acid sites of Ga/H-ZSM-5 collectively catalyze the dehydrogenation, oligomerization, and cyclization of olefins for aromatic substances, but inhibit the cracking reaction *via* Ga/H-ZSM-5 treated under different conditions with a reaction time of 10 h, as summarized in Fig. 7. Treatment conditions clearly affect the selectivity for propene, paraffin, and light olefins, as well as H_2_ and CO, but strongly affect propane conversion and BTX selectivity. In addition, the reduction-oxidation treatment of Ga/H-ZSM-5 (Ga/H-ZSM-5-RE-OX) prior to the reaction has a decisive effect on the ODP process and shows high propane conversion and selectivity for light olefins, paraffins, and the aromatic selectivity^47^ of the cracked products in the presence of CO_2_ compared to its absence, as shown in Fig. 6b and 7. This may be closely related to the position of the gallium species in the Ga/H-ZSM-5. However, the gallium species in the air-oxidized sample are likely to be present as finely dispersed Ga_2_O_3_ particles on the outer surface of the zeolite crystal, whereas the H_2_-reduced sample consists mainly of Ga^+^ and GaH^2+^. The effect of hydrogen is clearly to increase the dispersion of the gallium modifier, give rise generating more dual active sites combining intimately gallium species [(Ga^3+^, O^2−^) ion pairs)] and Brønsted sites from the zeolite, enhancing overall catalytic performance, and dispersed gallium species acting as portholes to release dihydrogen, thus reducing methane production and increase aromatic production.^48^ In contrast, the gallium species in the reduced and then oxidized sample is GaO^+^, which is located on the inner surface and the cation exchange state of ZSM-5.^49^ It has been reported that the initial aromaticity activity of GaO^+^ is much higher than that of Ga^+^ and GaH^2+^.^50^ In addition, well-dispersed GaO^+^ present in zeolite channels may allow for close contact with propane and intermediates. Therefore, Ga/H-ZSM-5-RE-OX, in the presence of CO_2_, represents an increased catalytic activity for aromatization of propane and light olefin selectivity compared to the absence of CO_2_ (Ga/H-ZSM-5-RE-OX). As evident, Zr–Ga/H-ZSM-5 at 550 °C shows high propane conversion and propene selectivity in the presence of CO_2_ compared to in its absence (58% *vs.* 53%, and 25% *vs.* 21%), as can be seen in Fig. 6c and 7. The Zr modification of the Ga/H-ZSM-5 zeolite resulted in stronger metal–support interactions between the Ga species and the zeolite.^51,52^ According to NH_3_-TPD studies,^53^ it was suggested that the zirconia supplementation provides weaker acidic sites (Lewis acid site) and more active sites for catalysis, as shown in Fig. 2 and Table 2. Furthermore, excess zirconia prevents the loss of activation of gallium species during the reaction by providing electrons to anchor the active gallium species. Therefore, these phenomena were responsible for the high catalytic activity of the Zr–Ga/H-ZSM-5 catalyst in terms of target selection.^54–56^ Furthermore, the addition of zirconium increased the gallium dispersion and prevented gallium crystallization, according to the XRD analysis (Table 4). Based on the above, there are three reasons for the improvement in the activity of the Ga/H-ZSM-5 catalyst achieved by zirconium doping. First, the introduction of zirconium promotes gallium dispersion and prevents gallium crystallization. Second, the addition of zirconium is beneficial for the enrichment of gallium species on the surface of H-ZSM-5 grains, facilitating energy and mass transfer in the presence and absence of the CO_2_ during the ODP process. Third, the presence of gallium and zirconium doping plays an important role in the formation of the Ga and Zr mixed oxides. This greatly increases the oxygen content of the reactive lattice and contributes to the formation of Zr^3+^ ions or oxygen vacancies around Zr^4+^ ions, improving the redox potentials of Zr–Ga/H-ZSM-5. The oxygen vacancies allowed for a relatively free way for the oxygen atoms to approach the gallium atoms. H_2_-TPR analysis for the Zr–Ga/H-ZSM-5 catalyst (see Fig. 3 and Table 3) shows that the introduction of zirconium leads to lower temperature at the main reduction peaks. In addition, significant hydrogen consumption at about 650 °C leads to the reduction of gallium ions produced in the zirconium oxide lattice, resulting in a stronger interaction between gallium and zirconium.^57^ The product distribution for propane conversion by Zr–Ga/H-ZSM-5-RE-OX during the ODP reaction in the presence and absence of CO_2_ is shown in Fig. 6c and 7. When dehydrogenation was performed in the absence of the oxidizing agent (CO_2_), the selectivity for methane was relatively high, as shown in Fig. 7. This indicates that oxygen was consumed by the catalyst during the initial process, resulting in oxygen vacancies formed during the initial step that could not be replenished because no oxidizing agent was available in the gas stream. This is in agreement with the previous literature.^58^Fig. 6d shows higher propane conversion and propene selectivity in the presence of CO_2_ than in its absence (61% *vs.* 59%, and 23% *vs.* 18%) at 550 °C over Cr/Zr–Ga/H-ZSM-5. The reason for this is that Cr species were reduced from Cr^6+^ to Cr^3+^ (or Cr^2+^) in Zr–Ga/H-ZSM-5 due to the Cr on the Zr–Ga/H-ZSM-5 catalyst being very stable to hydrogen treatment (H_2_-TPR analysis; Fig. 3 and Table 2) and not being oxidized to a higher oxidation state by calcination in air. However, the reduced Cr species on the H-ZSM-5 are readily reoxidized by treatment to Cr^6+^ (or Cr^5+^) variants. Then, the activation energy returns to approximately the same initial value as for CO_2_ treatment after hydrogen treatment.^59^ This suggests that reduced Cr active species can be readily reoxidized by CO_2_ to a more active initial state, which is consistent with the work of Ohishi *et al.*^60^ and Takehira *et al.*^61^ studying Cr-MCM-41 catalysts for the dehydrogenation of ethylbenzene to styrene and propane to propylene. They studied the Cr species on MCM-41 mesoporous silica and concluded that a redox process involving carbon dioxide-forming Cr^6+^ species is involved in the dehydrogenation. The product distribution for propane conversion over Cr/Zr–Ga/H-ZSM-5-RE-OX during the ODP reaction in the presence and absence of CO_2_ is summarized in Fig. 6d and 7. The highest dehydrogenation activity was found for the ZrO_2_-supported chromium catalyst as the chromium oxide present on the zirconia surface is stabilized in its highly dispersed state.^62^ The turnover frequency (TOF) representing the activity of H-ZSM-5 zeolites doped with Ga, Zr, and Cr for the ODP reaction, as carried out at 400–550 °C at atmospheric pressure in the presence and absence of CO_2_, is also shown in Table 4 and Table S-2 (ESI†). Here, the TOF is defined as the number of moles of propane/product that reacted or were produced per unit mole of acid sites per unit time. The amount of acid was estimated using NH_3_-TPD, as listed in Table 2. It can be seen that the Cr/Zr–Ga/H-ZSM-5 catalyst has the highest TOF_C_3_H_8__ compared to other catalysts, which can be ascribed to the high Ga content's dispersion on the catalyst surface, in accordance with the XRD results (Table 4). In particular, for all catalysts at the start of the reaction, the propane conversion was higher in the absence of CO_2_ than in its presence, as can be seen in Fig. 6, Table 4, and Table S-2.† This is probably indicative of CO_2_ being absorbed on the metal oxide-dopped H-ZSM-5 catalyst surfaces and/or on the active sites, forming carbonate species at low temperatures and initially blocking the sites to propane adsorption at (above 550 °C) corresponding to the reaction temperature. However, with the desorption of CO_2_ above 550 °C resulted from decomposition of carbonate species and the thermal reduction of metal oxides, with the latter becoming the active species with lower activity towards dehydrogenation of light alkane and being responsible for the catalytic behavior in the ODP reaction.^63^ Although nanoparticles of oxides with basic properties could be active for dehydrogenation of hydrocarbon at low temperatures, the strong adsorption of olefins on active sites make the reaction impracticable.^64^ For ODP-CO_2_, the strong adsorption of H_2_O formed during oxidation could adsorb on active sites.^65^ Thus, reactants and products in ODP-CO_2_ contribute to blocking the basic active sites at low temperatures. Both effects result in a decrease in the initial conversions in the presence of CO_2_.^20^ The catalyst deactivated much more slowly in the presence of CO_2_ than in its absence of CO_2_. This, in the presence of CO_2_, is due to the elimination of coke *via* Boudouard's reaction (CO_2_ + C → 2CO) and its transformation into H_2_ into CO and H_2_O through the RWGS reaction, as well as sustainably supplementing the reducible oxygen species on the surface.^66^ Therefore, the application of the bare and Ga-, Zr-, and Cr-doped H-ZSM-5 zeolites, as well as ODP-CO_2_ in non-equilibrium, increased CO_2_ dissociation and limited coke deposition due to C_3_H_8_ decomposition. Due to the lower dissociation energy of C_3_H_8_ compared to that of CO_2_, and because free radical processes predominate in ODP-CO_2_ reactions, the conversion of C_3_H_8_ was also greater than that of CO_2_, as shown in Fig. 6 and Table 5. Therefore, it is suggested that the coke on the bare H-ZSM-5 zeolite and those doped with Ga, Zr, and Cr was still mainly formed due to C_3_H_8_ decomposition on the catalyst surface. In addition, the effect of CO_2_'s partial pressure on promoting dehydrogenation of propane depends on the type of catalyst and the type of metal used. For easily oxidized metals such as Ga, Zr, and Cr, CO_2_ mainly reoxidizes the reduced active species in the reaction process, thereby leading to an increase in performance of the process. Lower initial activity in the presence of CO_2_ can also lead to inhibition of carbon formation on the surface of the catalyst. This agrees with the results presented in Fig. 8. The trend for CO_2_ conversion was more stable than for the propane conversion for all the H-ZSM-5 zeolites doped with Ga, Zr, and Cr under otherwise identical conditions, as shown in Table 5. Furthermore, carbon balances reach even higher levels: close to 86% (and possibly up to 100%), resulting in a loss of less than 14%. We suggest that the incomplete balances are caused mostly by the creation of H_2_O and the sum of less abundant (oxygenated) hydrocarbons and atom hydrogen that were not calibrated on the GC. Furthermore, carbon losse could be caused by coke deposition.^27^

**Table 5:** Summary of catalytic testing of metal-doped H-ZSM-5 zeolites in the presence of CO_2_

Catalyst	Conversion (mol%)			Selectivity (mol%)								TOF^a^_C_3_H_8__ (h^−1^)
	Temp. (^o^C)	C_3_H_8_	CO_2_	C_1_	C_2_	C_2_=	C_3_=	C_4_=	C_5_=	BTX	Carbon balance%	
H-ZSM-5	400	16	8	8	—	34	25	14	2	15	98	—
	450	25	16	10	—	26	22	9	5	26	98	—
	500	29	21	15	1	19	21	7	3	31	97	—
	550	34	25	18	1	10	20	3	1	35	88	—
4% Ga/H-ZSM-5	400	32	18	—	—	2	60	10	1	25	98	35
	450	35	23	5	—	7	37	15	2	31	97	36
	500	46	32	6	1	4	26	17	2	37	93	44
	550	54	42	9	1	2	25	8	1	40	86	49
4% Zr–Ga//H-ZSM-5	400	38	22	—	—	38	9	16	9	28	100	41
	450	44	28	—	—	25	17	19	2	37	100	44
	500	51	37	3	—	18	20	13	2	42	98	48
	550	58	46	5	1	10	25	7	—	45	93	51
1% Cr/4% 3Zr–Ga/H-ZSM-5	400	49	32	—	—	—	70	—	—	30	100	130
	450	53	45	—	—	2	52	3	—	40	97	131
	500	57	54	2	2	2	33	6	1	47	93	132
	550	65	61	4	2	1	23	7	1	54	92	141

a Estimated by moles of propane/product reacted or produced over per unit mole of acid cite per unit time.

**Table 6:** Summary of weight loss of metal-doped H-ZSM-5 zeolites in the presence and absence of CO_2_ at 800 °C

Catalyst	Coke content^c^ (wt%)	
	*C* a	*C* b
H-ZSM-5	12.21	8.01
4% Ga/H-ZSM-5	5.6	4.8
4% Zr–Ga/H-ZSM-5	6.8	3.9
1% Cr/4% Zr–Ga/HZSM-5	8.3	4.1

a In the absence of CO_2_.

b In the presence of CO_2_.

c Determined at 10 h time-on-stream.

#### 3.2.2. Propane dehydrogenation towards BTX

In a fixed bed reactor operating at atmospheric pressure, catalytic performance was tested. Fig. 6 and 7 showed the distribution of hydrocarbons, propane conversion, and BTX selectivity. The figures demonstrate that the aromatization of propane is a multi-step process that involves oligomerization, isomerization, cracking, and cyclization at Brønsted acid sites *via* a carbenium mechanism as well as dehydrogenation and/or hydrogen transfer across the Lewis acid sites. Dehydrogenation of propane to produce propene, oligomerization of the resulting alkenes, subsequent cracking (further conversion of the alkene), formation of new oligomers through alkenes alkylation, hydrogen transfer, dehydrogenation, cyclization, and aromatization are the steps involved in the conversion of light alkanes to aromatics. These steps are performed in this order. One of the most crucial phases in the aromatization process is alkane dehydrogenation because it initiates the reaction steps upon which subsequent reaction is influenced.^67^ According to Fig. 8, the presence of surface hydrogen caused oligomerized alkanes to fracture into lighter alkanes, resulting in poor BTX selectivity for bare HZSM-5. Ga-, Zr-, and Cr-impregnated H-ZSM-5 catalysts reduced cracking on H-ZSM-5, which increased the synthesis of BTX compounds by dehydrogenation, a process facilitated by metal oxides in recombining surface hydrogen produced. This is consistent with the fact that the acidity of the dehydrogenating metal modified H-ZSM-5 affects the catalytic conversion of alkanes in heterogeneous catalytic process and the related selectivity towards BTX. This conclusion is consistent with the NH_3_-TPD profile seen in Fig. 2 and Table 2. Propane conversion to BTX compounds requires strong acid sites of HZSM-5 and Lewis acid sites aided by adding metal oxides for dehydrogenation steps of surface hydrogen generated.^68^ With the aid of the addition of metals, the NH_3_-TPD peaks displayed decreased Brønsted acidity and increased Lewis acidity, increasing selectivity for BTX and stabilizing the reaction.^19^ Ga–, Zr–, and Cr– were present, which resulted in a consistent product distribution (BTX) with less lighter gases produced as a consequence of decreased cracking due to the presence of Ga-, Zr-, and Cr-active sites for surface hydrogen recombination. The greatest selectivity and product distribution toward BTX were provided by Cr/Zr–Ga/H-ZSM-5. This could be due to well dispersion on the zeolite network and interaction between Ga–, Zr–, and Cr–.

#### 3.2.3. Coke formation

The catalyst entered a period of steady operation, where propene, light olefins, and BTX selectivity were high and carbon deposition was very low in either the presence or absence of CO_2_. During the aromatization process, the larger molecules mainly occupy the acid sites and thereby block the micropores, this results in coke formation and subsequently catalyst deactivation.^69^ Hence, it is important to compare the carbon deposition of spent catalysts over bare H-ZSM-5 catalyst, Ga, Zr, and Cr modified ZSM-5 in the presence or absence CO_2_. These data in Fig. 8 and Table 6 imply that dopants metals, treatment conditions prior to reaction, and CO_2_ addition are likely responsible for reducing carbon content and hence increasing PDH activity. This is compatible with CO_2_-TPD profiles (as shown in Fig. S-1, ESI†), because basicity of the catalyst was revealed to influence carbon deposition, with a higher number of basic sites resulting in less carbon deposition. The increased basicity enhances CO_2_ activation on the surface of the catalyst, which combines with carbon produced as a result of side reactions. As a result, the reverse Boudouard reaction (2CO ↔ CO_2_ + C) transforms the carbonaceous species into CO. As a result, the catalyst with the highest basicity is projected to have the least amount of carbon deposition.^70^ The results of quantity coke analysis of spent catalysts in absence of CO_2_ are gathered in Table 6 and Fig. 8. Around 2.5 wt% of coke was measured over the bare H-ZSM-5 spent catalyst. Ga-, Zr-, and Cr-containing spent catalysts in absence CO_2_ showed higher levels of coke formation than the results in presence CO_2_. 5.6 wt% of coke was seen over the Ga/H-ZSM-5 spent catalyst. More coke was observed on the spent catalysts of Zr–Ga/H-ZSM-5 (6.8 wt%) and Cr/Zr–Ga/ZSM-5 (8.3 wt%), respectively. This behavior can be attributed to differences in acidity associated with assumption that high acid density sites (as shown in Table 2) facilitate the aromatization process for the production of aromatic products which can then be converted into coke species. This variance in coke generation from the produced catalysts may be mostly explained by changes in acidity (see Fig. 2 and Table 2) and pores structure (microporous and microporous mesoporous framework).

## 4. Conclusion

Herein, we reported the preparation ZSM-5 zeolite with various metal dopants using the wet impregnation technique. The catalytic behavior of these catalysts, which was evaluated according to the oxidative dehydrogenation of propane in the presence and absence of CO_2_, have been presented. The characterization of the various catalysts showed that the physiochemical properties of the H-ZSM-5 were modified by incorporation of metal dopants. The catalytic results indicated that the product distribution varied with the type of metal dopant used, and that propane conversion was dependent on the acidity of the catalyst in the presence of CO_2_. NH_3_-TPD, and catalytic evaluation demonstrated that the metal-doped H-ZSM-5 is weakly to moderately strongly acidic, which explains its superior catalytic performance. Unlike the lower methane ethylene selectivity, the amount of propene and BTX compounds were enhanced in the presence of CO_2_ for all metal-doped H-ZSM-5 catalysts. It seemed that the high dispersion of gallium species played a decisive role the in the light alkane dehydrogenation reaction. Framework Al atoms of HZSM-5 were found to be very useful for diffusing Ga_2_O_3_ because of their strong interactions with GaO_x_ particles, which could increase the latter's apparent Lewis acidity, as well as their ability to activate the C–H bond of propane. Cr and Zr have been identified as active CO_2_ activation sites. They are, indeed, electron-rich species capable of donating electrons to the CO_2_ antibonding orbitals, weakening the C–O bond and promoting the process. As an added bonus, the presence of the promoter resulted in the presence of oxygen atoms, which could aid in the gasification and removal of carbon deposits from the alkane decomposition.

## Data availability

All data generated or analyzed during this study are included in this published article’ in the main manuscript.

## Conflicts of interest

The authors declare no competing financial interest.

## Supplementary Material

RA-013-D2RA08235G-s001
