# Supplementary material for: Analysis and process evaluation of metal dopant (Zr, Cr)-promoted Ga-modified ZSM-5 for the oxidative dehydrogenation of propane in the presence and absence of CO2
Source: RSC Adv. 2023 Apr 6;13(16):11081–95. doi: 10.1039/d2ra08235g (PMC10077947; doi:10.1039/d2ra08235g)
Supplement: RA-013-D2RA08235G-s001 [file RA-013-D2RA08235G-s001.pdf]

Electronic Supplementary Material (ESI) for RSC Advances.
This journal is © The Royal Society of Chemistry 2023

Supplementary information

Analysis and Process Evaluation of Metal Dopant (Zr, Cr)-Promoted Ga-Modified Zeolite-5 for the Oxidative Dehydrogenation of Propane in the Presence and Absence of CO_2

Abbas Jawad[a*], and Sura Ahmed[b]

Midland Refineries Company MRC/AL Daura Refinery Company /Training and Development Division/ Baghdad, Iraq[a]

ajd5d@mst.edu

Midland Refineries Company MRC/AL Daura Refinery Company/Maintenance Board/Baghdad, Iraq[b]

bearn_bearn2020@yahoo.com

Table S-1: Elemental composition of metal oxides derived from XRF analysis.

Composites	Si/Al	Ga (wt%)	Zr (wt%)	Cr (wt%)
H-ZSM-5	49.89	-	-	-
4%Ga/H-ZSM-5	49.56	4.01	-	-
4%Zr-Ga/H-ZSM-5	49..21	3.96	4.06	-
1%Cr/4%Zr-Ga/H-ZSM-5	48.96	3.95	3.94	1.02

Table S-2: Summary of catalytic test of metal doped-H-ZSM-5 zeolites in absence CO_2

Catalyst	Temp. (^oC)	Conversion (mol%) C_3H_8	Selectivity (mol%) C_1	C_2	$C_2^=$	$C_3^=$	$C_4^=$	$C_5^=$	BTX	TOF^a_{C3H8} (h^{-1})
H-ZSM-5	400	13	15		40	19	11	6	9	
	450	22	21		30	16	10	4	19	
	500	23	25	1	28	12	7	4	23	
	550	32	27	2	33	9	6	6	27	
4%Ga/H-ZSM-5	400	28	4		5	65	7	1	18	31
	450	33	6	2	8	49	8	1	26	34
	500	38	8	3	9	28	19	3	30	37
	550	48	11	4	7	23	17	2	36	43
4%Zr-Ga//H-ZSM-5	400	34		1	39	6	25	2	27	36
	450	42	2	1	33	11	19	1	33	42
	500	49	5	1	27	17	10	2	38	45
	550	53	7	3	19	21	6	2	42	46
1%Cr/4%Zr-Ga/H-ZSM[illegible]	400	45			2	60	6		32	119
	450	51	1	1	4	43	14	1	36	126
	500	55	4	1	5	26	20	3	41	127
	550	59	6	2	4	18	17	3	50	128

[a] Estimated by moles of propane/product reacted or produced over per unit mole of acid cite per unit time.

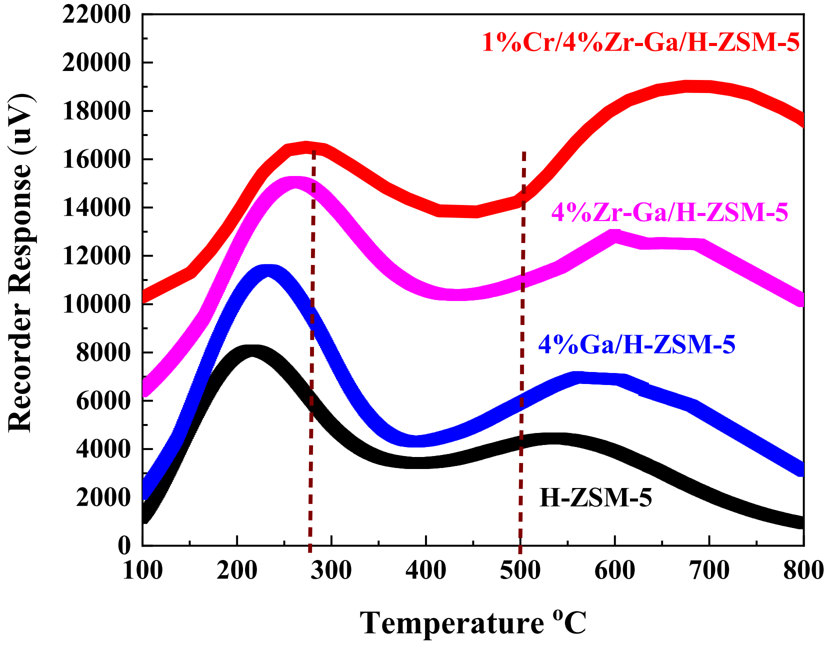

Fig. S-1. CO_2-TPD profiles of the bare and Ga-, Zr-Ga-, and Cr/Zr-Ga-doped H-ZSM-5.
